# Numerical Modeling of Phase Transformations in Dual-Phase Steels Using Level Set and SSRVE Approaches

**DOI:** 10.3390/ma14185363

**Published:** 2021-09-17

**Authors:** Krzysztof Bzowski, Łukasz Rauch, Maciej Pietrzyk, Marcin Kwiecień, Krzysztof Muszka

**Affiliations:** Faculty of Metals Engineering and Industrial Computer Science, AGH University of Science and Technology, al. Mickiewicza 30, 30-059 Kraków, Poland; lrauch@agh.edu.pl (Ł.R.); mpietrz@agh.edu.pl (M.P.); mkwiecie@agh.edu.pl (M.K.); muszka@agh.edu.pl (K.M.)

**Keywords:** phase transformations, dual-phase steels, SSRVE, level set method, continuous annealing

## Abstract

Development of a reliable model of phase transformations in steels presents significant challenges, not only metallurgical but also connected to numerical solutions and implementation. The model proposed in this paper is dedicated to austenitic transformation during heating and ferritic transformation during cooling. The goal was to find a solution which allows for the decreasing of computing time without noticeable decreasing the accuracy and reliability of the model. Proceedings to achieve this goal were twofold. Statistically Similar Representative Volume Element was used as a representation of the microstructure. It allowed for the reducing of the complexity of the computational domain. For the purpose of the model, carbon diffusion was assumed to be the main driving force for both transformations. A coupled finite element–level set method was used to describe growth of a new phase. The model was verified and validated by comparing the results with the experimental data. Numerical tests of the model were performed for the industrial intercritical annealing process.

## 1. Introduction

Research has been carried out for years on the development of steels with a multiphase microstructure. The design of optimal process parameters, e.g., temperature, time, and cooling rate, is possible thanks to simulations, in which numerical models of phase transitions are used to determine the correlation between parameters of technological processes and changes in a steel’s microstructure and its properties. Numerous models for predicting the kinetics of phase transitions and resulting microstructure and properties can be found in the literature. These models are characterized by different predictive capabilities and different computational complexity; see review in [1]. Depending on their construction, material models are divided into two groups today: mean-field and full-field models. In the former, the microstructure is implicitly represented by equations describing average parameters of the microstructure. In the latter the microstructure is represented explicitly in a geometrical form using Representative Volume Element (RVE) or Digital Materials Representation (DMR) concepts (e.g., see [2]). This approach is classified as Integrative Computational Materials and Process Engineering (ICMPE), see [3] for details. Typically, due to their high computing costs, the full-field models with complex functionality are not useful for the simulation of technological processes. This practically eliminates the possibility of using mathematically advanced and complex models in industry, where the time of conducted calculations is as important as the reliability of obtained results. On the other hand, simplified mean-field models do not meet the increasingly demanding requirements. Thus, the main goal of the present work was to search for a balance between computing costs and predictive capabilities of the models and to create a bridge between fast mean-field and advanced full-field models. Two actions were taken to achieve this goal. In order to meet the requirement of low computation times and maintain reasonable accuracy of the model, it was decided to use the concept of Statistically Similar Representative Volume Elements (SSRVEs) proposed in [4]. This allowed for the reducing of the complexity of the computational domain. In the model itself, a coupled finite element (FE) and level set (LS) method was used. This coupling allowed reliable and reasonably fast predictions of the motion of the interface.

The developed model was then integrated with an optimization procedure to select temperature cycle parameters for selected industrial processes. Finally, the model was verified against experimental data to confirm the validity of the presented solution.

## 2. Review of the Phase Transformation Models

In engineering calculations, where, primarily, the speed of calculations is crucial and models are mainly expected to provide basic information such as the contribution of individual phases or transformation kinetics, models using the JMAK (Johnson–Mehl–Avrami–Kolmogorov) equation [5,6,7] are used. A more physical model of the transformations was proposed by Leblond and Devaux in [8], who demonstrate numerous problems with the applicability of previous models under varying thermal conditions. Despite its simplicity, the model shows satisfactory predictive capabilities for a wide range of temperature change rates. The model using control theory equations [9] is an extension of the ideas of the Leblond model. The primary motivation of the authors of the model [9] was the premise that the kinetics of the phase transformation is similar to the response of a second-order inertial element in control theory. The concept of the model is to use a differential equation to describe the transition between two states of phase equilibrium.

The full-field models based on virtual representation of the microstructure are discrete models, differing mainly in the way the microstructure itself is represented, i.e., the description of grain boundaries. In the group of these models a distinction is made between implicit and explicit approaches. The explicit models capture the microstructure in an unambiguous way, assigning each point of the computational space to a specific grain and phase. This group includes models based on cellular automata [10,11] and front tracking methods. In implicit models, grains are represented by a field function, and the boundary between grains is the area in which the function changes or takes on assumed values. The group of these models includes solutions based on the phase-field method or the level set method [12].

Another group of models used in modeling microstructure changes are models with a diffusive interfacial boundary, in which the phase separation does not occur stepwise, but is continuous over some narrow area. In conventional techniques for modeling phase transitions and microstructure evolution, the boundary between different phases is assumed to be sharp. This means that the change in material properties occurs as rapidly as the position of the boundary. For each region, a set of differential equations is solved to obtain the distribution of the desired variable (e.g., element concentration). In addition, it is often necessary at the boundary to derive a boundary condition in the form of mass flux and to take steps to ensure the truth of the constitutive equations of thermodynamic equilibrium.

The group of diffusive boundary models includes those based on the phase-field method and the level set method. Due to its efficiency and reliability, the latter approach was used in the present work.

RVEs (Representative Volume Elements), although by definition they should represent the smallest acceptable elementary cells, are in many cases still too large to be effectively used in numerical calculations. For complex grain morphologies of multiphase structures, elementary cell determination and representativeness assessment are often time consuming or even impossible. Thus, as has been mentioned in the introduction, statistical simplification of the RVE was used in the present work to reduce the complexity of the computational domain.

**Figure 1:**
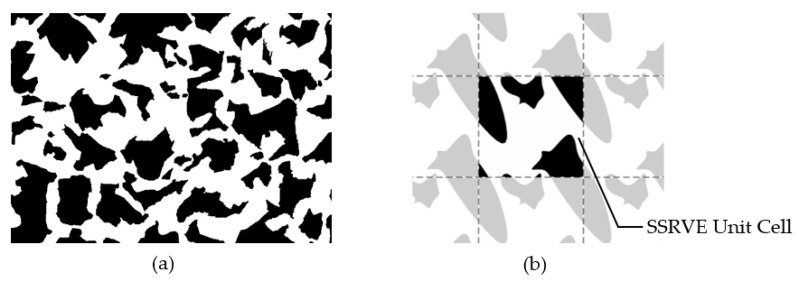
The idea of the statistically similar representative element. (**a**) Reference microstructure image and (**b**) periodic SSRVE [16].

The use of statistical analysis to model the effect of microstructure morphology on material properties using RVEs is not a new concept [13,14]. Balzani et al. in [4] proposed the creation of an artificial periodic elementary cell that could replace the representative element of an RVE in a statistically similar manner. The idea behind Statistically Similar Representative Volume Elements (SSRVEs) is to minimize the difference between selected measured parameters of a reference microstructure and an artificially generated elementary cell. By definition, the cell size should be significantly smaller than the size of the reference microstructure image (Figure 1). Authors developed an algorithm for generation of the SSRVE for dual-phase steels [15]. This algorithm was adapted in the present work to reproduce a ferritic–pearlitic microstructure prior to annealing and to create a computational domain for the simulation of phase transformations.

## 3. Methodology of Determining SSRVEs

The determination of the Statistically Similar Representative Volume Element (SSRVE) cell is a typical optimization problem. In each iteration of the optimization procedure, the shape and size of the grain (or grains) in the SSRVE cell are modified. The minimized objective function is the mean squared error between the reference values and the estimated values for the analyzed shape in a given iteration:

(1) $$\frac{1}{k}\left[\sum_{i=1}^{k}w_{i}{\left(\zeta_{i}^{SSRVE}-\zeta_{i}^{REF}\right)}^{2}\right]+\varepsilon \to \min,$$

where: $\zeta_{i}^{REF}$—reference coefficient value, $\zeta_{i}^{SSRVE}$—coefficient value for the SSRVE cell, *w_i_*—coefficient weight, and *ε*—penalty function value. The penalty function includes checking the shape for intersecting segments.

**Figure 2:**
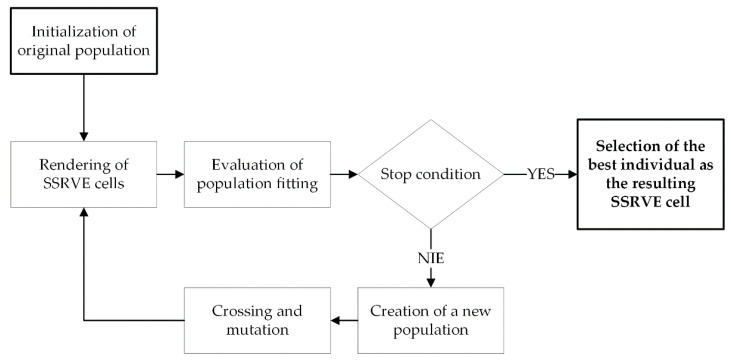
SSRVE generation workflow.

The basic workflow of SSRVE generation is shown in Figure 2. In this paper, NURBS (Non-Uniform Rational B-Spline) curves are used to create shapes of artificial grains. A NURBS curve is defined by a vector of control points, a vector of nodes and the degree of the curve. The control points determine the shape of the curve, the nodes determine the influence of each control point on the curve, and an appropriate sequence of nodes produces closed curves. The optimization procedure used is based on a modified genetic algorithm [17].

## 4. Phase Transformation Model

### 4.1. Level Set Method (LSM)

The level set method (LSM) belongs to the group of implicit methods for tracking the interfaces. This means that the boundary is not given directly by a set of points or curves, but is the result of the evaluation of some function. The LSM method was developed by Osher and Sethian [18], and has been appreciated in diverse problems of modeling the motion of sharp boundaries. The versatility of this technique has also been noted in modeling phenomena occurring within the microstructure of metallic materials, as evidenced by work in modeling grain growth [19] or recrystallization of polycrystalline materials [20].

LSM involves separating the computational space into two regions, i.e., positive and negative values of certain function *φ*. The region (zero level set) where the two regions meet determines the phase boundary. Each region represents a separate phase or separate material property. The mathematical form of the partition boundary, Γ, in the level set approach can be written as follows:

(2) $$\Gamma =\left\{x\in \Omega |\phi (x)=0\right\},\;\phi :\Omega \subset {\mathbb{R}}^{n}\to \mathbb{R}.$$

**Figure 3:**
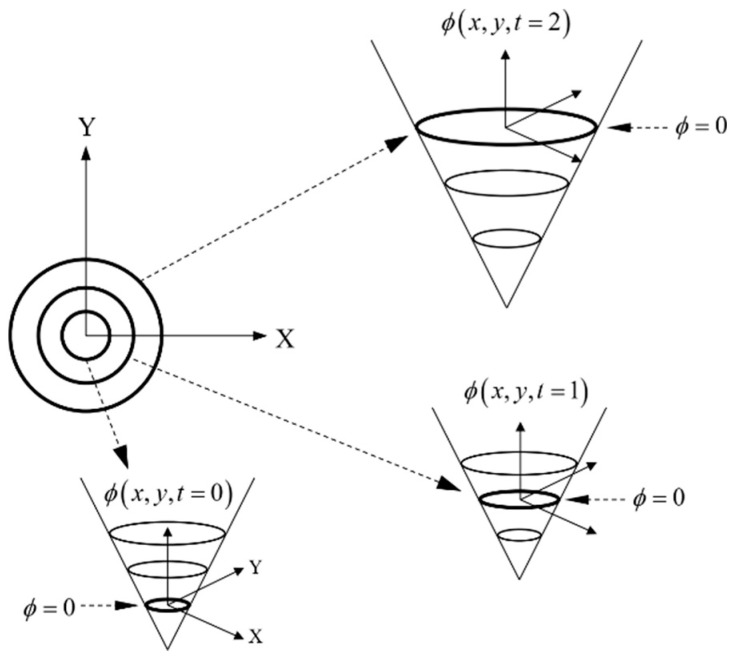
Boundary evolution scheme using the level set method.

The final form of the level set equation takes the form:

(3) $$\frac{\partial \phi }{\partial t}+F\left|\nabla \phi \right|=0,$$

where the field function, *F*, defines the velocity of the boundary depending on external and internal forces. Solving Equation (3) allows the position of the boundary at successive time steps to be obtained, as schematically shown in Figure 3.

From a numerical point of view, Equation (3) belongs to the group of hyperbolic advection equations, and its numerical solution requires additional steps to obtain numerical stability without oscillations. For this purpose, an artificial diffusion or viscosity term is introduced into the equation, which acts only in the direction of motion [21]. Although the equation of levels is sufficient to solve the boundary motion problem alone, it requires the definition of the vector velocity field of the boundary migration. Therefore, in practical solutions, it is usually coupled with equations that allow the boundary velocity to be estimated directly, e.g., using the Navier–Stokes equation, or indirectly using the temperature or energy gradient.

**Figure 4:**
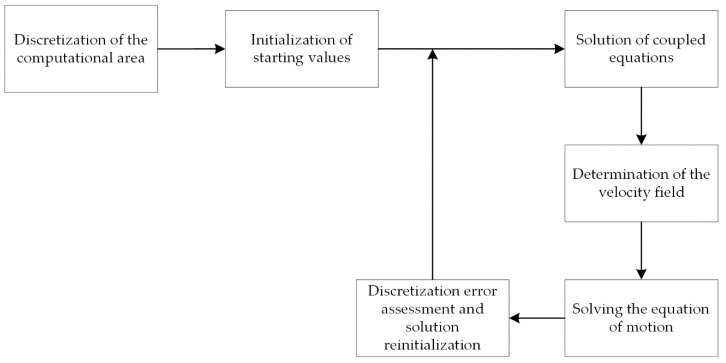
Diagram of the LSM algorithm.

The conventional solution to a problem using the LSM method involves iterative executing of a set of steps to evaluate the solution at each time step (Figure 4).

Initialization of the solution consists in applying a characteristic function to describe the region, Ω, in the first time step. The most common method used for initialization is a Signed Distance Function (SDF). Each degree of freedom in the system is assigned a value equal to the distance of a given point in the global system, to the nearest point on the boundary. Depending on the phase (area), the assigned value has a positive or negative sign. By definition, points lying on the boundary take the value 0.

(4) $$\begin{array}{l} \phi \left(x\right)=\{\begin{array}{cc} +d\left(x\right) & x\in \Omega^{+}\\ 0 & x\in \Gamma \\ -d\left(x\right) & x\in \Omega^{-} \end{array}\\ d\left(x\right)=\min_{x_{0}\in \Gamma }\left|x-x_{0}\right|. \end{array}$$

Velocity in problems of boundary motion is usually a relation arising directly or indirectly from the physical basis of the phenomenon or the properties of the medium. In most numerical problems, the velocity of the boundary can be determined only directly at the boundary itself, but the differential nature of the motion function makes it necessary to perform an extrapolation of the velocity vector to the entire computational region. This procedure is called velocity extension in level set method terminology. Commonly, the fast marching method [22] is used.

With the velocity field defined, the boundary motion problem can be solved. Once converted to the weak formulation, the differential equation is solvable by conventional computational methods, such as the finite difference method or the finite element method. In this paper, a method for solving Equation (3) is based on application of a diffusion term and Streamline Upwind Petrov–Galerkin (SUPG) stabilization [23]. In this method, the weight function is defined in the form:

(5) $$W_{I}=N_{I}+\frac{\alpha h}{2}\frac{u_{k}}{\left|u\right|}\frac{\partial N_{I}}{\partial x_{k}},$$

where: *N_I_*—standard interpolation function in the Galerkin method, *h*—characteristic dimension of the element, *u_k_*—advection velocity, and *α*—weight parameter of SUPG scheme. The final system of equations takes the form:

(6) $$M\dot{\phi }+K\phi =0$$

where **M** and **K** matrices take the following forms:

(7) $$\begin{array}{l} M=\int \left(N_{I}N_{J}+\frac{\alpha h}{2}\frac{u_{k}}{\left|u\right|}\frac{\partial N_{I}}{\partial x_{k}}N_{J}\right)d\Omega \\ K=\int \left(N_{I}u_{p}\frac{\partial N_{J}}{\partial x_{p}}+\frac{\alpha h}{2}\frac{u_{k}}{\left|u\right|}\frac{\partial N_{I}}{\partial x_{k}}u_{p}\frac{\partial N_{J}}{\partial x_{p}}\right)d\Omega \end{array}$$

where: *I*, *J*—1, …, number of degrees of freedom; *k*, *p*—1, …, number of dimensions.

According to the Courant–Friedrichs–Lewy (CFL) condition for explicit schemes [19], the time step, Δ*t*, in the time discretization must satisfy the condition:

(8) $$CFL=\frac{\left\|u\right\|\cdot \Delta t}{h}\leq 1$$

The calculations assume a time integration scheme:

(9) $$\phi_{j}^{n+1}={\left(M+\Delta t\theta K\right)}^{-1}\left(M-\Delta t\left(1-\theta \right)K\right)\phi_{j}^{n}$$

where: *θ* = 0—Euler’s explicit scheme, *θ* = 1/2—Crank–Nicholson scheme, and *θ* = 1—implicit Euler scheme. The numerical models presented in this paper uses an implicit Euler scheme.

Stabilization of the equation with an artificial diffusion term leads to the loss of properties by the distance function with successive iterations of the algorithm. The solution quality degradation is also affected by the accumulation of numerical errors of the solutions. Both of these factors cause the zero level set to degrade, leading to distortion or stretching of the isolines and resulting in significant degradation of the solution. Therefore, it is necessary to periodically perform reinitialization of the solution (also known as redistancing), which consists in restoring the properties of the distance function without changing the position of the zero level. Reinitialization can be realized either by solving the differential equation [24] or by analytical methods. In the first case, it is necessary to solve the equation in the form:

(10) $$\phi_{t}=\mathrm{sgn}\left(\phi_{0}\right)\left(1-\left|\nabla \phi \right|\right)$$

with an initial condition as the current distribution of level sets, *φ*_0_. The solution is delivered in an iterative manner until a steady state is obtained in the entire computational domain of the solution, i.e., the condition is satisfied:

(11) |∇ϕ|=1

Equation (10), however, despite its relatively fast convergence, requires multiple solutions across the domain, which definitely increases the computation time. 

Good reinitialization results have been obtained using the so-called geometric method [25], which uses the properties of the FEM shape function to find the exact position of the zero level within the element discretizing the surface. For each element through which the zero level set passes, i.e., for each element for which the criterion is satisfied:

(12) $$\underset{\phi \in \Omega_{E}}{\forall }\min\left(\phi \right)\cdot \max\left(\phi \right)\leq 0$$

A set of points lying on the zero level set is determined. The shape functions of the element in the local coordinate system are used to estimate the coordinates of the positions where the boundary intersects the faces of the reference element. For example, in the case of a quadrilateral element with first-order interpolation, the used position of the points on the zero level set is interpolated in the local coordinate system of the element using:

(13) $$\begin{array}{l} \phi \left(\xi ,\eta \right)=\frac{1}{4}\left(1-\xi \right)\left(1-\eta \right)\Phi_{e}\left(-1,-1\right)+\frac{1}{4}\left(1+\xi \right)\left(1-\eta \right)\Phi_{e}\left(1,-1\right)\\ +\frac{1}{4}\left(1-\xi \right)\left(1+\eta \right)\Phi_{e}\left(-1,1\right)+\frac{1}{4}\left(1+\xi \right)\left(1+\eta \right)\Phi_{e}\left(1,1\right) \end{array}$$

The sampling density is controlled by the *n_p_* parameter.

(14) $$\begin{array}{ll} \left(\xi ,\eta \right)=\left(-1+\frac{2}{n_{p}}k,\eta \right) & k=0,1,\ldots ,n_{p}\\ \left(\xi ,\eta \right)=\left(\xi ,-1+\frac{2}{n_{p}}k\right) & k=0,1,\ldots ,n_{p} \end{array}$$

**Figure 5:**
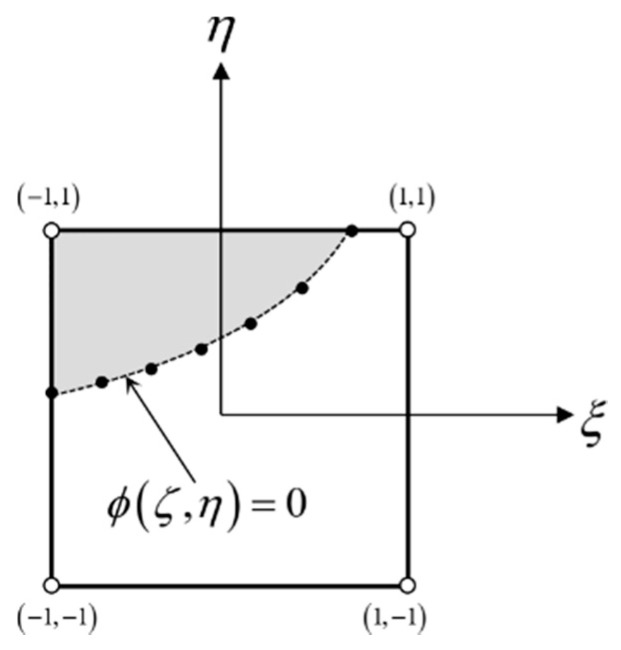
Finite element. The idea of boundary discretization in the geometric reinitialization method.

Then, for each degree of freedom, the algorithm reconstructs the correct distance by finding the closest boundary point. The idea of the geometric reinitialization method is shown in Figure 5.

In the presented method, the geometric method is further developed by introducing R-trees into the algorithm to support the search for points on the boundary in the reinitialization process. The key idea of R-trees is the introduction of bounding boxes, which allow quick estimations as to whether the searched value is in a given subtree. This significantly avoids unnecessary node visits while maintaining a well-balanced structure. 

### 4.2. MLS-DIFF Model

The MLS-DIFF (Multiple Level Set and Diffusion) model is a coupled solution using the formulation of the boundary motion problem (using the multi-level set method) and the diffusion of carbon, which is the driving force of the transformation. It is a development of the approach presented in [26]. The model was implemented using deal.II [27]. The boundary velocity is defined in terms of the classical relation [28]:

(15) v=MΔG

where: *M*—the mobility of the boundary defined in the form:

(16) $$M=M_{0}\exp\left(-\frac{Q_{M}}{RT}\right)$$

where: *M*_0_—mobility constant, *Q*_M_—activation energy, *R*—gas constant, and *T*—temperature. The driving force, Δ*G*, in equation (16) depends on the carbon concentration and takes the form:

(17) $$\Delta G=\chi \left(C_{\gamma \alpha }-C_{\gamma }\right)$$

where: *χ*—proportionality factor, *C_γα_*—equilibrium carbon concentration in austenite, and *C_γ_*—average carbon concentration in austenite. 

It is important to consider that the evolution of boundaries can lead to the merging of regions and the disappearance of grain boundaries. While such an assumption works if both regions have the same properties and, in fact, their merging leads to the formation of a coherent phase (e.g., in two-phase flow), it is completely unsuited to the polycrystalline microstructure, in which growing grains do not usually merge due to their different crystallographic orientations. When simulating the evolution of such a microstructure, it becomes important to preserve the morphology and size of individual grains, which the basic formulation of the method does not allow. 

This problem was solved by a modification of the method that introduces separate partial solutions, which are then combined into a final assembly [19]—the Multiple Level Set (MLS). In the present work, each modeled grain is assumed to be a separate region described by an individual level set function. The shape of the grain and the velocity of the boundary movement during the transformation are influenced by other grains with which it is adjacent. Although the motion of the boundary of all grains is computed on a single-grid basis, each grain has individually initialized degrees of freedom and velocity field. This approach to the problem makes it necessary to take into account grain collisions, i.e., situations in which growing grains that are independent functions come into contact, so that without interference they would occupy a single physical region. The additional correction step after each time step is introduced. If the value of a function at a given grid node is positive for more than one level set function, then all conflicting degrees of freedom are corrected using the following equation:

(18) $$\phi_{i}^{c}=\frac{1}{2}\left(\phi_{i}^{p}-\underset{i\neq j}{\max\left(\phi_{j}^{p}\right)}\right)$$

where: *φ_i_^c^*—value of the *i*-th level function for a given degree of freedom after correction and *φ_i_^p^*—value of the *j*-th level function before correction. The final grain morphology is obtained using a property of the distance sum function that preserves the position of all the boundaries:

(19) $$\tilde{\phi }=\underset{x\in \Omega }{\forall }\max\left(\phi_{i}\right)$$

where: *x*—degree of freedom position and *φ_i_*—value of level set function at a given position.

The phase transformation model presented in this work uses the MLS concept coupled to the FE solution of the carbon diffusion equation. The zero level position is not guaranteed at a grid node at each time step. This creates a challenge for coupled problems where a boundary condition is applied only at the boundary. Therefore, this paper employs *h*-adaptation, which involves local densification of elements through hierarchical partitioning.

The diffusive transport of a carbon is described by Fick’s second law in the form:

(20) $$\frac{\partial c}{\partial t}=D\nabla^{2}c$$

where: *D*—carbon diffusion coefficient, *c*—carbon concentration, and *t*—time.

Equation (20) describes the change in concentration of a component over time. The solution of Equation (20) is obtained using the finite element method. The value of the carbon diffusion coefficient depends on the temperature, type of crystal lattice, crystalline imperfections, and concentration of the diffusing substances. The exact value of the carbon diffusion coefficient in ferrite and austenite is the subject of much debate [29,30,31]. In this paper, the carbon diffusion coefficient in the austenite is assumed to be described by the Ågren equation [31], as it takes into account both the effect of carbon concentration and temperature. The carbon diffusivity is much higher in alpha iron due to the lower atomic packing and larger interstitial spaces compared to the gamma iron structure. The limited solubility of carbon in alpha iron makes the diffusivity significantly limited and negligibly small from the point of view of the numerical solution. Therefore, the diffusion of carbon in ferrite is neglected in the presented approach. The numerical solution of (20) reduces to forming and solving a system of linear equations of the form:

(21) $$\begin{array}{l} c_{j}^{{}^{n+1}}={\left(M+\Delta t\theta \cdot K\right)}^{-1}\left(M-\Delta t\left(1-\theta \right)\cdot K\right)c_{j}^{n}\\ M=\int \left(N_{I}N_{J}\right)d\Omega \\ K=D\int \left(\frac{\partial N_{I}}{\partial x_{i}}\frac{\partial N_{J}}{\partial x_{j}}\right)d\Omega \end{array}$$

together with the boundary and initial conditions derived from the simulation conditions. The solution results in the distribution of carbon concentration in austenite.

**Figure 6:**
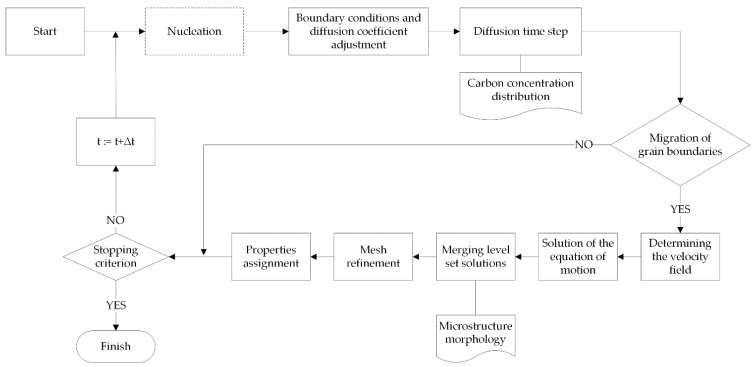
Scheme of the phase transformation model algorithm.

The flowchart of the algorithm for simulating phase transformations using the MLS-DIFF method is shown in Figure 6.

The individual steps of the algorithm are the same for both heating and cooling transformations, the difference being the nucleation step, which occurs only during cooling. In the heating simulation the nucleation stage is omitted and the initial microstructure is treated as austenitic–ferritic.

#### 4.2.1. Austenitic Transformation

Perlite dissolution and the austenite nucleation process are omitted, i.e., after the transformation starts each perlite grain is treated as a single austenite grain in the simulation. Based on the morphology of the initial microstructure, a single-carbon diffusion simulation is initiated. The initial condition for austenite grains in terms of carbon concentration is calculated from the eutectic point at temperature *A_e_*_1_. We assume that the functions describing carbon concentration in the austenite at the *γ*-*α* boundary (*C_γα_*) and at the *γ*-cementite boundary (*C_γβ_*) are described by the following relations:

(22) $$\begin{array}{l} C_{\gamma \beta }=a_{1}T+b_{1}\\ C_{\gamma \alpha }=a_{2}T+b_{2} \end{array}$$

The temperature, *A_e_*_1_, is the point of intersection of these functions:

(23) $$A_{e1}=\frac{b_{2}-b_{1}}{a_{1}-a_{2}}$$

The concentration at the eutectic point is obtained by substituting the temperature, *A_e_*_1_, into Function (22). Based on the current simulation time, the temperature is determined, from which the diffusion coefficient and the boundary motion condition are calculated. For simplicity, it is assumed that carbon diffusion occurs only in the austenite, which is dictated by the limited solubility of carbon in ferrite. The boundary motion condition for each grain is that the average carbon concentration at the boundary (*c*_Γ_) exceeds the maximum carbon content in the austenite at that temperature:

(24) $$c_{\Gamma }\left(T\right)>=C_{\gamma \alpha }\left(T\right)$$

The motion of the boundary entails determining the velocity field and then solving Equation (3) for each grain requiring adjustment of the boundary position. The evolution of a single grain involves the execution of the MLS algorithm for all the components of the level set functions (i.e., the correction and concatenation of the component functions). Local *h*-adaptation is performed at the grain boundary, followed by assignment of the properties of each grid’s cell to austenite or ferrite, depending on the value of the level set functions after the evolution step. The stop condition of the algorithm is based on reaching the target temperature. 

#### 4.2.2. Ferritic Transformation

The allocation of the solution of the level function is done gradually in the nucleation stage. In this paper, it was decided to use the nucleation model [32] which allows the nucleation velocity, *I_v_*, to be estimated in the form:

(25) $$I_{v}=N_{\mathrm{het}\;}v\exp\left(\frac{-\Delta G^{*}}{kT}\right)\exp\left(\frac{-E_{M}}{RT}\right)$$

where: *v*—Debye frequency (~10^13^ s^−1^), *k*—Boltzmann constant, *T*—temperature, *R*—gas constant, *E_M_*—activation energy of interfacial diffusion, *N_het_*—density of nucleation sites, and $\Delta G^{*}$—the nucleation barrier energy that must be overcome to produce an embryo of critical size. The nucleation barrier energy is determined from the relationship:

(26) $$\Delta G^{*}=\frac{\Psi }{\Delta g_{V}^{2}}$$

where: Ψ—a factor capturing the difference between the energy of the native phase and the nucleus boundary and the shape of the nucleus ($2.1\times 10^{-6}J^{3}/m^{6}$) [33], and $\Delta g_{v}$—nucleation momentum (difference in Gibbs free energy per unit volume between the parent phase and the forming phase).

Density of nucleation sites is the potential number of favored sites (edges, corners, and triple points) per unit volume. This value depends primarily on the grain size of the parent phase (austenite). Cahn [34] proposed for its description a relation in the form:

(27) $$N_{het}=n_{v}{\left(\frac{\delta }{d_{\gamma }}\right)}^{3-v}$$

where: *n_v_*—number of carbon atoms per unit volume, *ρ*—effective grain boundary thickness ($2.5\times 10^{-10}\;m$), *d_γ_*—grain diameter of the parent phase, and *v*—factor capturing the dimension of nucleation sites (homogeneous nucleation—three, along the grain boundary plane—two, along grain edges—one, and at triple points—0).

**Figure 7:**
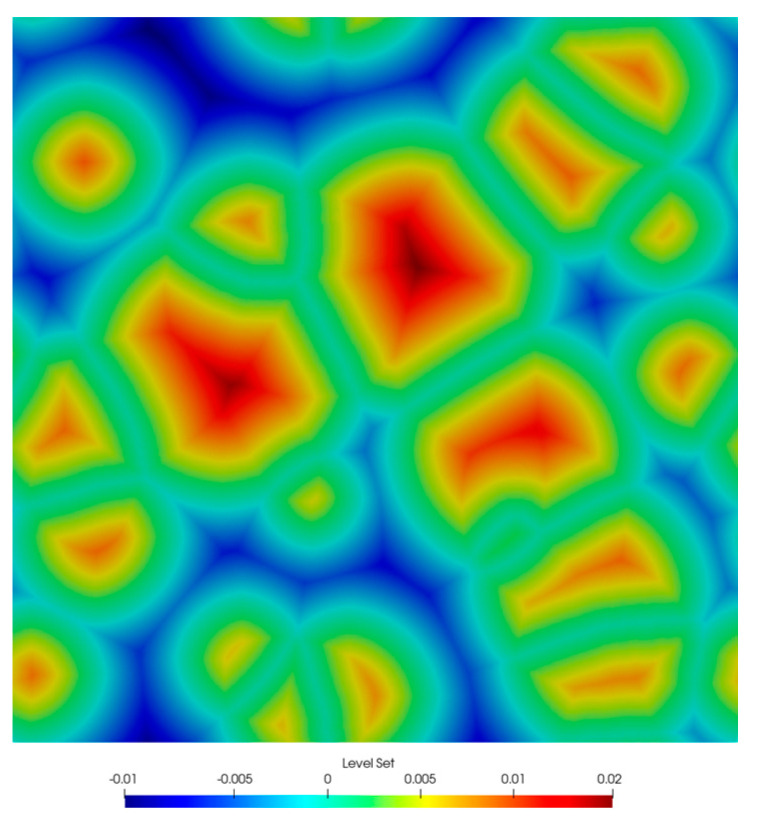
Image of microstructure described by level set functions.

Temperature for the current simulation time is calculated based on the assumed thermal cycle. Depending on the temperature, both the diffusion coefficient and the boundary condition are determined. At each time step of the main loop of the algorithm, the nucleation rate and the number of active nuclei in the computational domain are calculated. If this quantity exceeds the number of active nucleation points, new ones are activated in a fixed order. Each new nucleus translates into the activation of a new solution of the level set function initialized with the distance function, such that positive values fall inside the ferrite nuclei. The solutions are then combined according to the typical MLS approach described earlier. An example microstructure image representing the sum of the individual level set functions is shown in Figure 7.

It is assumed that new grains have the shape of circles with a given radius. In each time step, at the boundary *α*/*γ*, the equilibrium concentration of carbon is given equal to *C_γα_*(*T*) in the form of the Dirichlet condition. The concentration value at the boundary is calculated from the GS line of the equilibrium diagram (the line below which austenitic transformation begins). The initial condition for the diffusion simulation is a given carbon concentration in the austenite region equal to the average carbon content of the steel. The concentration distribution can be homogeneous or heterogeneous, depending on the assumptions of the simulated process. After solving the diffusion equation, the mass conservation condition (carbon concentration), which is a predictor of the boundary motion, is checked:

(28) $$\int_{\Omega_{\gamma }}\left(c(x)-c_{0}\right)d\Omega_{\gamma }>\int_{\Omega_{\alpha }}\left(c_{0}-c_{\alpha }\right)d\Omega_{\alpha }$$

If the condition is not satisfied, the next diffusion time step is calculated. If boundary motion is required, the velocity field for each of the ferrite grains is determined, and then the MLS equation is solved and the separate solutions are merged. Similarly to the austenitic transformation, cells are assigned to ferrite or austenite depending on the sign of the function value. At the boundary, *h*-adaptation is performed to reduce the discretization error for the boundary condition. 

## 5. Model Verification

Two low-carbon steels, A and B, whose chemical compositions are given in Table 1, were selected for the study. The first belongs to typical structural steels of general purpose, and it was used for model validation and verification using microscopic examination. The latter, which belongs to the dual-phase (DP) group of advanced high-strength steels, was used to show the model’s capability to simulate industrial processes.

### 5.1. Laboratory Verification

**Figure 8:**
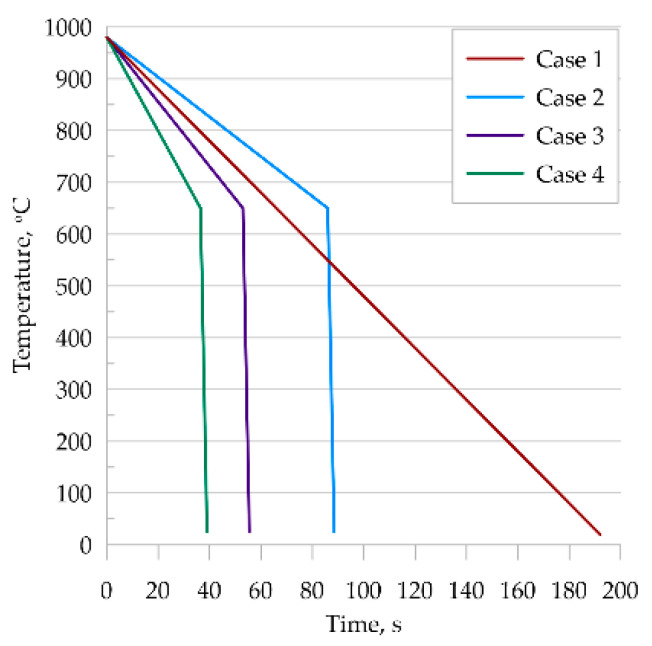
Temperature cycles used.

In this study, four thermal cycles were considered (Figure 8), which were reproduced in the dilatometric tests.

**Figure 9:**
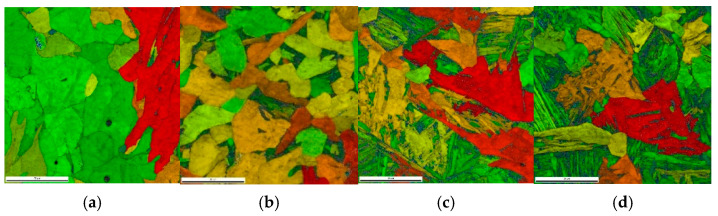
EBSD results for (**a**) case one; (**b**) case two; (**c**) case three; and (**d**) case four.

- Each of the selected temperature cycles was mapped using the MLS-DIFF model. For this purpose, a digital representation of a 100 × 100 μm-base microstructure with an average grain size of 30μm was prepared and simulations were performed on it. The initial microstructure is ferritic–perlitic. Obtained results from the EBSD analysis are presented in Figure 9. It can be seen that in the case of thermal cycle one and two, diffusive transformation products were obtained (with equiaxed ferrite morphologies), while in the case of thermal cycle three and four, acicular ferrite and bainite were produced, respectively.

**Figure 10:**
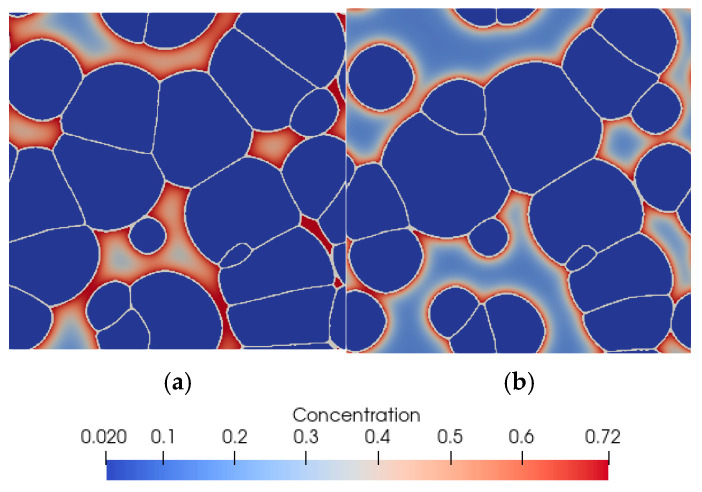
Final microstructure morphology (ferrite) predicted by the model with carbon concentration distribution in martensite for (**a**) case one and (**b**) case two.

Example results obtained from the model for case one and case two are presented in Figure 10.

**Figure 11:**
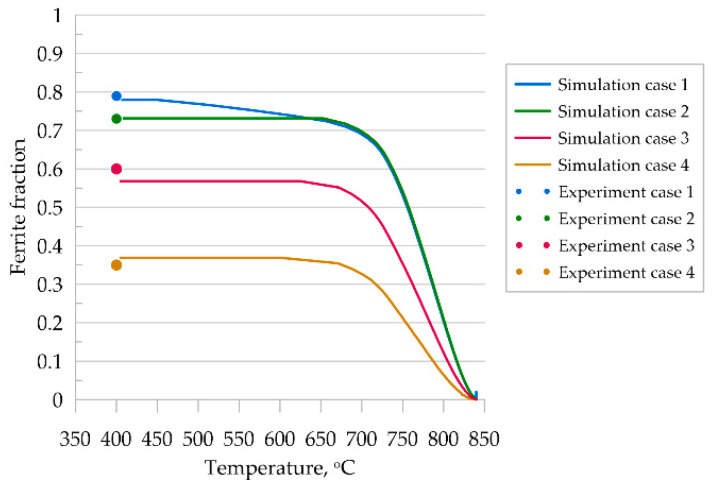
Kinetics of ferritic transformation according to MLS-DIFF model, comparison to experimental results.

The analysis of the obtained results shows a satisfactory agreement of the presented model with experimental results for steel A. For cases three and four, the grain morphology is very difficult to compare qualitatively due to the irregular grain shape. High cooling rates of the samples resulted in non-diffusive growth of ferrite and consequently obtained needle-shaped grains, which the presented diffusion model was not able to reproduce faithfully. A comparison of the quantitative results is shown in Figure 11. It can be seen that final ferrite fractions were correctly predicted for all thermal cycles.

**Figure 12:**
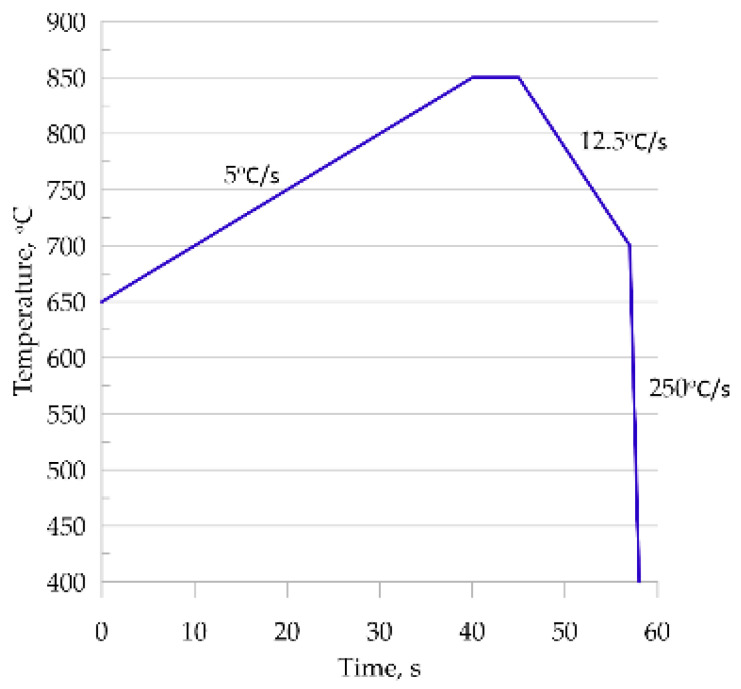
The heating and cooling cycles used in the simulations.

To show the predictive capabilities of the developed and validated model, it was used to simulate an industrial process. Intercritical continuous annealing was selected as an example [35]. A comparison of phase transformation simulation results for the RVE and SSRVE was performed on steel B. A simplified continuous annealing cycle shown in Figure 12 was considered. The thermal cycle included heating and cooling stages, and it was reproduced for testing purposes. 

**Figure 13:**
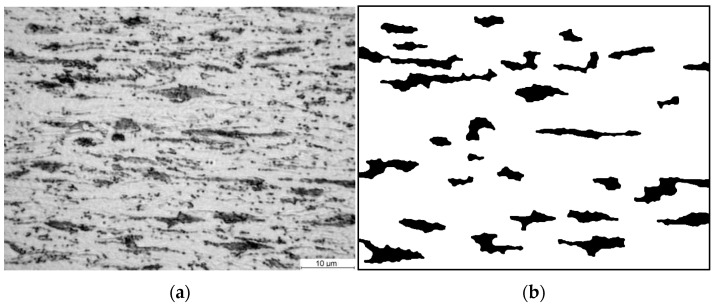
(**a**) Photo of the microstructure of steel B in the cold-rolled condition and (**b**) image of the ferritic–perlite microstructure after image postprocessing.

Figure 13a shows the microstructure of cold-rolled DP steel strip with a total reduction level of 74%. This microstructure was an input for the simulations of the continuous annealing. The microstructure shows perlite grains, with a characteristic elongated morphology resulting from deformation by rolling, in a ferrite matrix. 

**Figure 14:**
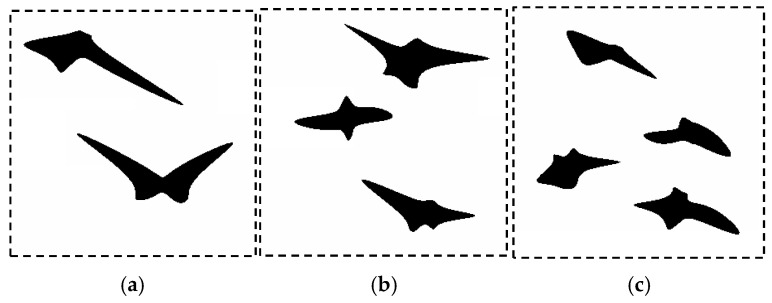
Examples of the SSRVEs based on the microstructure of B steel with: (**a**) two, (**b**) three and (**c**) four grains.

The photograph (Figure 13a) was subjected to a postprocessing procedure to obtain an unambiguous grain separation (Figure 13b). The prepared photo was analyzed to find representative coefficients. Based on the reference data prepared in this way, an optimization procedure was executed. The selection of the coefficients of the objective function was also motivated by the needs that coefficients must faithfully represent the elongated nature of the perlite grains. The unit cell size was 20 µm. The following coefficients were used in the objective function (1) of the SSRVE procedure: the Danielson coefficient, roundness II, ellipse fit, LP3, and phase fraction. SSRVEs with two, three, and four elements were prepared and are shown in Figure 14. Detailed description of each coefficient can be found in [35].

**Figure 15:**
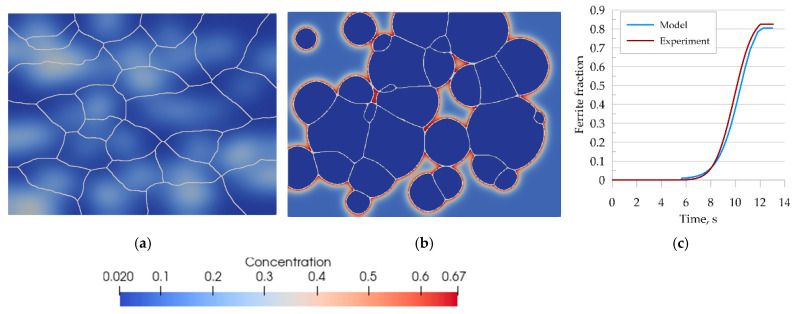
Morphology of the microstructure of steel B and carbon concentration distribution at (**a**) 870 °C, (**b**) after cooling to room temperature, and (**c**) the kinetics of ferritic transformation—comparison of the model with the experimental results.

The first verification step consisted of a full austenitizing simulation using a ferritic–pearlitic microstructure representation followed by cooling. Thus, the simulation included heating and cooling cycles shown in Figure 12. The results obtained from the simulation are shown in Figure 15.

The transformation kinetics upon cooling were compared with the experimental results and good agreement was obtained (Figure 15c). The error of curve fitting was estimated to be 3%. 

**Figure 16:**
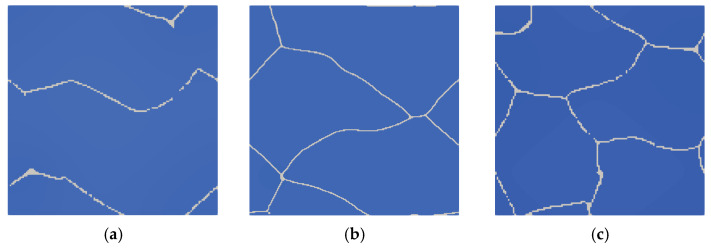
Simulation of phase transformation upon heating using SSRVEs with two (**a**), three (**b**), and four (**c**) grains.

Simulation of austenitic transformation using an SSRVE with two grains (Figure 16a) showed that the resulting morphology consists of infinitely long grains without triple points. The reason for this condition is the use of periodic boundary conditions. Although nucleation of ferrite across grain boundaries is possible, the banded microstructure would not reflect the actual microstructures of DP steels. Three-element and four-element SSRVEs were used for cooling simulations (Figure 16b,c). 

**Figure 17:**
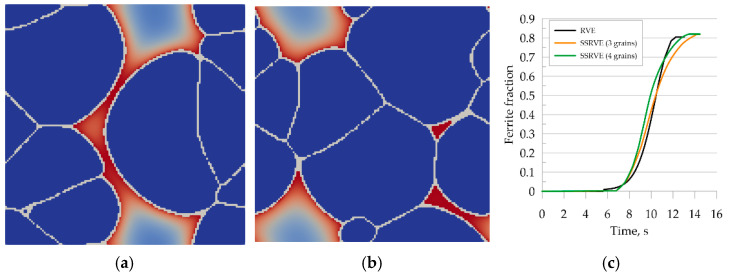
Simulation of the phase transformation under cooling using SSRVEs, with three perlite grains (**a**) and four grains (**b**). Comparison of transformation kinetics obtained for SSRVEs and the RVE (**c**).

Good agreement of the quantitative results is observed in Figure 17c, where a comparison of transformation kinetics obtained for SSRVEs and the RVE is shown. The final ferrite content predicted by the model is comparable for the RVE and SSRVEs. The slight differences in the transformation kinetics are caused, probably, by the limited number of nucleation sites or the lack of consideration of periodicity in the nucleation model. The mean square error between the transformation kinetics curves for SSRVEs and the RVE were 0.04 and 0.05 for elements with three and four inclusions, respectively. In our opinion, both of the presented SSRVEs can replace the more complicated RVE in the simulation of phase transformations.

### 5.2. Industrial Verification

**Figure 18:**
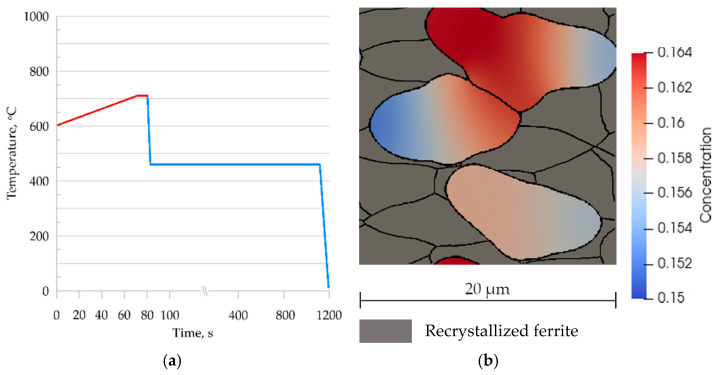
(**a**) Temperature cycle of continuous annealing; (**b**) microstructure morphology of steel B at 810 °C and carbon concentration distribution in the austenite grains.

Intercritical annealing is a heat treatment process in the AC1 and AC3 temperature range without full austenitization. The MLS-DIFF model allows the simulation of such a process. For this purpose, the temperature cycle shown in Figure 18a was adopted and used to perform a coupled heating and cooling simulation. Figure 18b shows the microstructure morphology at 810 °C obtained from a numerical simulation. About 50% ferrite remains in the microstructure at this stage, confirming report [35].

**Figure 19:**
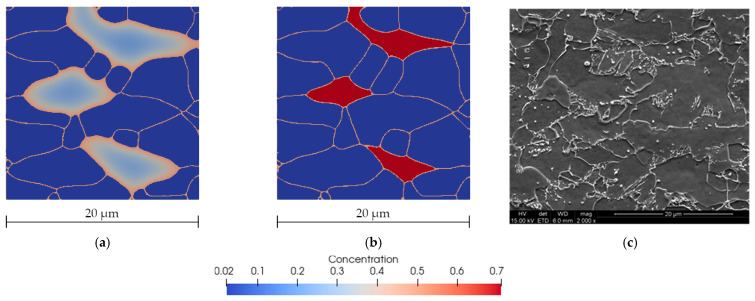
Microstructure morphology and carbon concentration distribution predicted by the model at 450 °C after 80 s (**a**) and at the end of the cooling cycle (**b**). SEM photography after the same cycle (**c**) [35].

Figure 19 shows the carbon concentration and microstructure morphology during and at the end of the cooling cycle. The image of the obtained microstructure was compared with the microscopic observations. It can be observed that the concentration of the carbon obtained from the computer modeling at the end of the cooling cycle is significantly higher which is consistent with metallurgical observations. Typical shapes of grain morphology were correctly reproduced. 

The presented results are consistent and the characteristic features of the microstructure were well-highlighted by the model, which is reflected in the microscopic images. The model predicted the final ferrite content of the microstructure to be 79%, which is close to experimental results [35] that indicated a value of 80%.

## 6. Conclusions

A new model based on the coupling of the FE solution of diffusion equations with the LS method was developed. The strength of the model is its applicability with limited material information. The model requires only key properties based on a phase equilibrium diagram to work properly. Known limitations of the presented model include limited predictive capability at rapid cooling where carbon diffusion is not the dominant driving force.

The model has been verified with experimental tests performed on low-carbon steels, with satisfactory results. An SSRVE was chosen as the method to simplify the microstructure of the material. It allows the complicated morphologies of the steel microstructure to be replaced by periodic cells statistically similar to their reference prototypes. Although the procedure for creating an SSRVE is computationally intensive, obtaining a representative element significantly reduces the time of further simulations. Consider the typical case of phase transformations—an RVE discretized with 200,000 finite elements required nearly 6 days to simulate a cooling cycle (real time 15 s). An SSRVE of the same element size could be simulated in less than 24 h. Tests were performed on a 16-core processor using all available compute threads. It is possible to design complex cycles of laminar cooling and continuous annealing by using the presented model. The application of such a solution allows not only the desired ratio of individual phases to be obtained, but also the grain size or element concentration gradient. It should be noted, however, that the calculation time in such a case is relatively long and it may become necessary to use distributed processing.

The selection of the shape coefficients used in the SSRVE generation procedure was preceded by sensitivity analysis based on histogram distribution. The coefficients with the leading expected values were selected. Although the methodology for their selection has been extensively tested, the nature of the study, based on image analysis, means that their final selection may be influenced by many factors, including a reference image that is not representative or inaccuracy of grain representation after image postprocessing. 

The selection of the number of grains in the SSRVE used in the simulation of phase transformations should be based on the analysis of the number and distribution of triple points of the austenite microstructure and the grain size. Further research in this area is necessary.

The significantly smaller number of finite elements used in the discretization of the SSRVE reduced the computational time substantially. It can be assumed that the computation time decreased proportionally to the reduction in the number of elements. The final computation time is also significantly affected by applied code parallelization methods.

The presented solution, through a loose coupling of two models, allows for a prospective further development of the method by taking into account the influence of the concentration distribution of other elements or by taking into account the deformation state. 

## Figures and Tables

**Table 1:** Chemical composition of the materials used in the simulations.

Steel	C	Al	Cr	Cu	Mn	Mo	Ni	Si	N	P	S
A	0.13	0.003	0.06	0.27	0.47	0.02	0.09	0.16	0.009	0.018	0.025
B	0.09	0.053	0.35	0.03	1.42	0.005	0.01	0.1	0.009	0.011	0.01
